# System-Level Hardware-Waveform Co-Design for Micro-UAV Radar Sensors: Suppressing Noise Folding in Extreme SWaP-C SSDBF Arrays

**DOI:** 10.3390/s26144574

**Published:** 2026-07-19

**Authors:** Xinhua Dai, Kai Xie, Youkang Wang

**Affiliations:** School of Electronics and Communication Engineering, Sun Yat-sen University, Shenzhen Campus, No. 66 Gongchang Road, Guangming District, Shenzhen 518107, China; daixh5@mail2.sysu.edu.cn (X.D.); wangyk58@mail2.sysu.edu.cn (Y.W.)

**Keywords:** spread spectrum digital beamforming (SSDBF), micro-UAV radar, remote sensing sensors, hardware-waveform co-design, noise folding, extreme SWaP-C constraints

## Abstract

Micro-Unmanned Aerial Vehicles (micro-UAVs) require compact radar arrays under severe Size, Weight, Power, and Cost (SWaP-C) constraints. Spread Spectrum Digital Beamforming (SSDBF) reduces receiver hardware by multiplexing multiple antenna channels before a shared RF chain, but the receiver-side switching operation folds wideband noise and injects switch-related transient noise before digital demultiplexing. This paper develops a hardware-waveform co-design framework that links the transition density of bipolar spreading sequences to high-frequency code energy and switching-event count, while explicitly distinguishing modulation-induced thermal-noise folding from switch-transient injection. Transition density is used as a physically interpretable design surrogate rather than a sufficient statistic for folded noise, and it is constrained jointly with non-zero-shift cross-correlation to preserve spatial isolation under receiver-side timing skew. An ϵ-constrained Greedy Coordinate Space Search (ϵ-GCSS) algorithm is proposed to synthesize low-transition-density SSDBF code sets. Commercial circuit-level transient simulations and system-level MATLAB R2024b simulations show that the proposed code set reduces the PRN-like transition-density level from about 0.50 to about 0.19, lowers the circuit-simulation-derived integrated IF noise power by 9.63 dB relative to the Gold/PRN-like baseline, and improves the normalized maximum detection range by 8.2 percentage points at K=4 without adding analog front-end hardware.

## 1. Introduction

The paradigm shift toward extreme Size, Weight, Power, and Cost (SWaP-C) platforms, such as lightweight UAV remote sensing platforms and micro-UAV swarms [1], imposes severe payload constraints on onboard radar sensors. High-resolution sensing, interference rejection, and multi-beam operation are most naturally supported by digital beamforming (DBF), because DBF preserves element-level spatial degrees of freedom (DoF) [2,3]. However, a conventional full-digital array requires one radio-frequency (RF) receive chain and one high-speed Analog-to-Digital Converter (ADC) for each antenna element. The corresponding power consumption, heat dissipation, routing density, and front-end volume are difficult to accommodate on micro-UAV radar payloads [4,5]. Hybrid beamforming reduces part of the digital burden, but it still typically relies on element-level active front-end components and phase-shifter networks, which remain costly under tightly packed Ku-band or millimeter-wave array spacing.

Spread Spectrum Digital Beamforming (SSDBF) provides an alternative hardware-reduction route [6,7,8,9,10,11]. Instead of assigning one complete RF chain to each antenna, SSDBF applies internal spreading codes to multiple receive channels and combines them before a shared chain. The digital back-end then demultiplexes the coded mixture and reconstructs the spatial channels. In this paper, *K* denotes the RF-link multiplexing depth within a subarray. The approach can reduce ADCs and downstream digital I/O while retaining the possibility of recovering the *N*-element spatial aperture [12,13,14].

This hardware efficiency is obtained at a physical cost. In the extreme SWaP-C topology considered here, the spreading operation is performed before the shared LNA, mixer, IF filter, and ADC. The received noise at the antenna ports is, therefore, multiplied by the switching codes before bandlimiting, and the RF switches themselves may add transient charge injection, clock feedthrough, and finite-transition artifacts. These effects should not be treated as ordinary downstream receiver noise: they occur before digital recovery and can raise the effective noise floor seen by every recovered spatial channel.

The absence of an element-level LNA or a high-Q preselector before the multiplexer is an engineering assumption that reflects the most constrained receiver case. At Ku-band array spacings, high-Q cavity filtering and per-element active gain are often incompatible with the volume, insertion-loss, and power budgets of a micro-UAV payload. This does not imply that all pre-filtering is infeasible in every implementation; rather, it motivates the LNA-less and preselector-limited topology as a stringent boundary case in which waveform-level mitigation becomes necessary. Under this topology, suppressing the spectral and transient content generated by the multiplexing codes is a direct way to reduce the analog impairment before it is amplified and digitized.

The central idea of this work is to redesign the internal SSDBF address codes according to hardware-aware criteria. Classical spread-spectrum sequences are usually selected for balance, pseudo-randomness, and bounded correlation. In the present receiver-side architecture, however, perfectly random toggling is not always beneficial. A high transition density increases the number of switching events and tends to spread code energy over a wider harmonic region. We, therefore, minimize the code transition density while enforcing a bounded non-zero-shift cross-correlation constraint to preserve spatial isolation under quasi-synchronous timing errors [15,16,17].

The main contributions are as follows:

1. Noise-folding model with separated physical mechanisms: We derive a receiver-side model that distinguishes modulation-induced thermal-noise folding from switch-transient noise injection. The model shows that the exact folded-noise level is governed by the code harmonic coefficients, while the transition density provides a tractable upper-bound metric for high-frequency code energy and a linear driver of switching-event count.
2. Jitter-aware sequence synthesis: We formulate a constrained binary code-set selection problem that jointly minimizes aggregate transition density and bounds worst-case shifted cross-correlation. The resulting ϵ-GCSS algorithm searches low-transition-density sequence families while maintaining spatial isolation under receive-chain timing skew.
3. System-level validation and range mapping: Using commercial circuit-level transient simulations and system-level MATLAB R2024b simulations, we compare Walsh, Gold/PRN-like, Kasami/length-compatible PRN, random binary, and proposed ϵ-GCSS sequence sets under identical SSDBF constraints. The reported 9.63 dB circuit-simulation-derived IF-noise reduction and 8.2-percentage-point range improvement are explicitly tied to the high-transition-density PRN baseline and the ideal zero-folding reference.

In this paper, bold uppercase letters (e.g., A) denote matrices, bold lowercase letters (e.g., a) denote vectors, and italic letters (e.g., a,A) denote scalars. Calligraphic uppercase letters (e.g., C,S) are used to denote discrete sets, with |S| representing the cardinality of the set. $R^{M\times N}$ and $C^{M\times N}$ represent the spaces of M×N real and complex matrices, respectively. Superscripts ${\left(\cdot \right)}^{T}$, ${\left(\cdot \right)}^{H}$, and ${\left(\cdot \right)}^{*}$ denote transpose, conjugate transpose, and conjugate operations, respectively. tr(·) and det(·) denote the trace and determinant of a matrix. ${∥\cdot ∥}_{2}$ and ${∥\cdot ∥}_{F}$ denote the Euclidean norm of a vector and the Frobenius norm of a matrix, respectively. The symbol E[·] denotes the statistical expectation operator, ⊗ denotes the Kronecker product, and $I_{N}$ denotes an N×N identity matrix. Additionally, CN(μ,Σ) denotes a complex Gaussian random distribution with mean μ and covariance matrix Σ.

The remainder of this paper is structured as follows: Section 2.1 establishes the extreme SWaP-C receiver topology and formulates the wideband noise-folding bottleneck. Section 3 develops the hardware-aware waveform optimization framework and the ϵ-GCSS algorithm under quasi-synchronous receiver-side timing-skew constraints. Section 4 presents the circuit-level and system-level validation results, including code-family benchmarking, timing-skew robustness, detection probability, and range recovery analysis. Section 5 discusses the applicability, limitations, validation scope, and scalability of the proposed framework. Finally, Section 6 concludes the paper.

## 2. Noise Folding Mechanism in SSDBF Receivers

### 2.1. System Model and Extreme SWaP-C Receiver Topology

**Figure 1:**
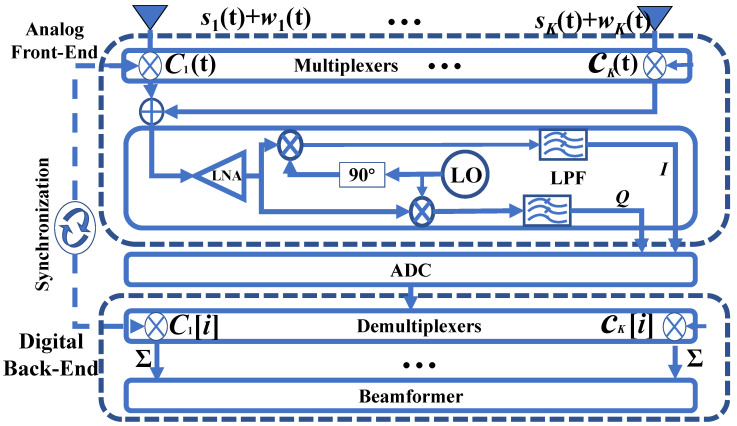
Extreme-SWaP-C SSDBF receive topology with analog code multiplexing before the shared RF chain. Here, $s_{n}\left(t\right)$ and $w_{n}\left(t\right)$ denote the received echo and antenna-port noise, respectively; $c_{n}\left(t\right)$ and $c_{n}\left[i\right]$ denote the analog spreading waveform and the corresponding digital despreading sequence; Σ denotes summation; and LNA, LPF, LO, I/Q, and ADC denote the low-noise amplifier, low-pass filter, local oscillator, in-phase/quadrature branches, and analog-to-digital converter, respectively. The dashed outlines identify the analog front-end and digital back-end functional boundaries.

The SSDBF receiver considered in this paper follows the multiplexer-first topology shown in Figure 1. This topology is adopted to represent the most constrained micro-UAV receiver case, where element-level active gain and high-order preselection are limited by power, routing, insertion-loss, and volume budgets. A uniform linear array (ULA) with *N* elements is partitioned into *M* subarrays, each connected to one shared RF chain. The RF-link multiplexing depth is

(1) $$K=\frac{N}{M}.$$

This partitioning is used for analog-domain code multiplexing rather than for analog beam steering. Its purpose is to aggregate *K* physical receive channels before the shared chain and then recover the *N* spatial channels after digital demultiplexing.

For a far-field narrowband target at angle $\theta_{0}$, the RF echo at the *n*-th element is

(2) $$s_{n}\left(t\right)=\sqrt{P_{r}}u\left(t-\tau_{0}\right)e^{j2\pi f_{c}t}e^{-j\frac{2\pi }{\lambda }d\left(n-1\right)\sin\theta_{0}},$$

where $P_{r}$ is received power, u(t) is the normalized baseband envelope, $\tau_{0}$ is the round-trip delay, $f_{c}$ is the carrier frequency, and *d* is the element spacing. The antenna-port thermal noise $w_{n}\left(t\right)$ is present before the multiplexing operation. This ordering is central to the present model: the noise is not added only after the shared receiver chain, but is first multiplied by the same receiver-side address code as the desired echo.

For the *m*-th shared chain, the connected element index set is $I_{m}=\left\{\left(m-1\right)K+1,\ldots ,mK\right\}$. Each channel is multiplied by a bipolar spreading waveform $c_{n}\left(t\right)\in \left\{+1,-1\right\}$ with chip duration $T_{c}$. The composite RF signal injected into the shared chain is

(3) $$y_{\mathrm{RF},m}\left(t\right)=\sum_{n\in I_{m}}\left[s_{n}\left(t\right)+w_{n}\left(t\right)\right]c_{n}\left(t\right)+q_{m}\left(t\right),$$

where $q_{m}\left(t\right)$ denotes switch-related transient injection. This term is written explicitly because it is physically different from the modulation of antenna thermal noise. The former is generated by the switching network itself through charge injection, control feedthrough, and finite transition effects, whereas the latter is the frequency translation of existing wideband antenna noise by the periodic code waveform.

After down-conversion and baseband filtering with equivalent impulse response $h_{B}\left(t\right)$, the multiplexed baseband signal can be written as

(4) $$y_{B,m}\left(t\right)\approx \sum_{n\in I_{m}}s_{B,n}\left(t\right)c_{n}\left(t\right)+v_{m}^{\left(\mathrm{th}\right)}\left(t\right)+v_{m}^{\left(\mathrm{sw}\right)}\left(t\right),$$

where $s_{B,n}\left(t\right)$ is the down-converted narrowband echo component. The two residual terms are

(5) $$\begin{array}{cc} v_{m}^{\left(\mathrm{th}\right)}\left(t\right)\; & =\left[\sum_{n\in I_{m}}w_{n}\left(t\right)c_{n}\left(t\right)e^{-j2\pi f_{c}t}\right]*h_{B}\left(t\right),\\ v_{m}^{\left(\mathrm{sw}\right)}\left(t\right)\; & =\left[q_{m}\left(t\right)e^{-j2\pi f_{c}t}\right]*h_{B}\left(t\right). \end{array}$$

Equation (5) makes the signal flow explicit: the shared filter acts on noise components that have already been modulated or generated before the digital demultiplexer. Therefore, even if the code set is orthogonal at zero shift, the filtered residual noise entering the digital correlator is colored by the analog switching and filtering cascade.

After analog-to-digital conversion, the *p*-th spatial channel is recovered by correlation with the corresponding local code over one code period:

(6) $${\tilde{r}}_{p}=\sum_{i=0}^{L-1}y_{B,m}\left[i\right]c_{p}\left[i\right]\approx L\sqrt{P_{r}}u\left(t-\tau_{0}\right)e^{-j\frac{2\pi }{\lambda }d\left(p-1\right)\sin\theta_{0}}+{\tilde{v}}_{p}.$$

Here, ${\tilde{v}}_{p}$ includes both folded thermal noise and switch-transient residuals after filtering and correlation. Equation (6) emphasizes the main receiver-side bottleneck: code orthogonality can recover the desired signal term and suppress ideal inter-channel interference, but it cannot remove noise that has already been translated into the shared IF/baseband passband before demultiplexing. This observation motivates the waveform-layer mitigation developed in the next section.

### 2.2. Wideband Noise Folding and Equivalent Cascade Model

For a periodic discrete code $c_{n}\left(t\right)$, the PSD can be expressed by harmonic lines

(7) $$S_{c_{n}}\left(f\right)=\sum_{k=-\infty }^{\infty }{\left|A_{n,k}\right|}^{2}\delta \left(f-k\Delta f\right),$$

where $\Delta f=R_{c}/L$, $R_{c}=1/T_{c}$, and $A_{n,k}$ is the *k*-th Fourier coefficient of the chip waveform. For an ideal rectangular-chip code, $A_{n,k}$ contains both the discrete Fourier coefficient of $c_{n}$ and the chip-shaping sinc factor. Therefore, the exact folded-noise level is not determined by a single scalar such as transition density; it is determined by the full set of harmonic powers $\left\{|A_{n,k}{|}^{2}\right\}$.

The spectrum of the modulated antenna thermal noise $w_{n}^{\prime }\left(t\right)=w_{n}\left(t\right)c_{n}\left(t\right)$ is

(8) $$S_{w_{n}^{\prime }}\left(f\right)=\sum_{k=-\infty }^{\infty }{\left|A_{n,k}\right|}^{2}S_{w_{n}}\left(f-k\Delta f\right).$$

After down-conversion and IF/baseband filtering, the thermal-noise component at the shared chain is

(9) $$S_{v_{m}}^{\left(\mathrm{th}\right)}\left(f\right)=\left[\sum_{n\in I_{m}}\sum_{k=-\infty }^{\infty }{\left|A_{n,k}\right|}^{2}S_{w_{n}}\left(f+f_{c}-k\Delta f\right)\right]{\left|H_{B}\left(f\right)\right|}^{2}.$$

The k=0 term is the in-band contribution, whereas k≠0 terms represent out-of-band noise translated into the IF/baseband band by the code harmonics.

The switching network also generates transient noise. Let $T_{n}=\left\{i:c_{n}\left[i\right]\neq c_{n}\left[i+1\right]\right\}$ denote the set of switching instants and let g(t) be the equivalent impulse response of one switching event, including charge injection, control feedthrough, and finite rise/fall effects. A behavioral representation of this contribution is

(10) $$q_{n}\left(t\right)=\sum_{i\in T_{n}}a_{i}g\left(t-iT_{c}\right),$$

where $a_{i}$ captures event polarity and device-dependent amplitude. Under uncorrelated or weakly correlated switching-event polarities, the average transient-noise PSD scales approximately as

(11) $$E\left\{S_{q_{n}}\left(f\right)\right\}\approx \left(L-1\right)\gamma_{n}\sigma_{a}^{2}{\left|G\left(f\right)\right|}^{2},$$

where $\gamma_{n}=\left|T_{n}\right|/\left(L-1\right)$ is the transition density. Thus, transition density enters the model in two ways: it upper-bounds the high-frequency harmonic energy of the code waveform and it linearly scales the number of switching events that excite device transients.

The channel-specific folding factor is defined as

(12) $$\begin{array}{cc} \alpha_{\mathrm{fold},n}\; & =\frac{\int_{-B_{\mathrm{IF}}/2}^{B_{\mathrm{IF}}/2}\sum_{k\neq 0}|A_{n,k}{|}^{2}S_{w_{n}}\left(f+f_{c}-k\Delta f\right){\left|H_{B}\left(f\right)\right|}^{2}df}{\int_{-B_{\mathrm{IF}}/2}^{B_{\mathrm{IF}}/2}|A_{n,0}{|}^{2}S_{w_{n}}\left(f+f_{c}\right){\left|H_{B}\left(f\right)\right|}^{2}df}\\ & \;+\frac{\int_{-B_{\mathrm{IF}}/2}^{B_{\mathrm{IF}}/2}S_{q_{n}}^{\left(\mathrm{sw}\right)}\left(f\right){\left|H_{B}\left(f\right)\right|}^{2}df}{\int_{-B_{\mathrm{IF}}/2}^{B_{\mathrm{IF}}/2}|A_{n,0}{|}^{2}S_{w_{n}}\left(f+f_{c}\right){\left|H_{B}\left(f\right)\right|}^{2}df}. \end{array}$$

The first term is modulation-induced thermal-noise folding, and the second term is switch-transient injection. This separation addresses the fact that both mechanisms are related to code switching but do not scale identically in real hardware.

Assuming an average folding factor ${\bar{\alpha }}_{\mathrm{fold}}$ over the selected code set, the equivalent noise factor of the SSDBF receiver is modeled as

(13) $$F_{\mathrm{eq}}=L_{\mathrm{mux}}\left[\left(1+{\bar{\alpha }}_{\mathrm{fold}}\right)K+\frac{F_{\mathrm{RF}}-1}{G_{\mathrm{ant}}}\right],$$

where $L_{\mathrm{mux}}$ is the insertion loss of the multiplexing network, $F_{\mathrm{RF}}$ is the noise factor of the shared RF chain, and $G_{\mathrm{ant}}$ is the available gain before the multiplexer. In the LNA-less topology, $G_{\mathrm{ant}}\approx 1$, so the folded-noise term has a direct impact on receiver sensitivity.

### 2.3. Power Budget Analysis and Waveform Objective

Table 1 provides a representative power comparison for a 32-element Ku-band micro-UAV sensor. The values are component-level estimates used to motivate the architecture, not a universal hardware specification. The key point is that sharing ADCs and digital I/O can save several watts, while adding per-element active gain or high-order preselection may erase a large part of this SWaP-C advantage.

The waveform objective is, therefore, to reduce ${\bar{\alpha }}_{\mathrm{fold}}$ without adding array-scaled analog hardware. Since (12) depends on the code harmonic distribution and (11) depends on the number of switching events, transition density is used as a hardware-aware design surrogate. It is not treated as a sufficient statistic for all folded noise; the final candidate sequences are also evaluated by PSD integration, cross-correlation, timing-skew robustness, and detection-range mapping.

## 3. Waveform-Layer Mitigation: Departing from Classical Spread Spectrum Under Hardware Constraints

The preceding section establishes where the sensitivity loss enters the SSDBF receiver: the analog codes affect both the harmonic distribution of antenna thermal noise and the number of switching events that excite device transients. The remaining design question is, therefore, not simply how to choose an orthogonal code set, but how to choose a code set that is simultaneously hardware benign, spatially separable, and robust to receiver-side timing skew.

Related hardware-aware radar waveform and discrete optimization frameworks motivate the use of constrained multiobjective and binary design strategies [18,19,20,21].

This section develops the waveform-layer design in three conceptual steps, followed by the offline search strategy used to construct the final code set. First, transition density is introduced as a physically interpretable surrogate for high-frequency code energy and switching-event count. Second, the ideal synchronized case is used to identify Walsh low-sequency selection as a useful zero-shift lower-bound reference for switching activity. Third, this ideal reference is extended to a quasi-synchronous receiver model, where shifted cross-correlation must be controlled because practical analog code application and digital demultiplexing are not perfectly aligned. Based on this progression, the ϵ-GCSS algorithm is then introduced as an offline search method that balances transition-density reduction and shifted-correlation survival. This organization preserves the original signal-processing structure while incorporating the hardware clarifications required for the revised model.

### 3.1. Transition Density as a Spectral-Energy Surrogate

Classical spread-spectrum and communication-oriented sequences are usually valued for balance, pseudo-randomness, and favorable correlation behavior. Such properties are desirable when the sequence is used as a transmitted spreading waveform or as a multiuser separation code. In the SSDBF receiver considered here, however, the code is an internal analog address tag that directly drives RF switching before the shared receiver chain. A rapidly toggling sequence may, therefore, be attractive from a conventional spread-spectrum viewpoint but unfavorable from a hardware-noise viewpoint.

For a bipolar code $c_{n}=\left[c_{n}\left[0\right],c_{n}\left[1\right],\ldots ,c_{n}\left[L-1\right]\right]$, $c_{n}\left[i\right]\in \left\{+1,-1\right\}$, the transition density is defined as

(14) $$\gamma_{n}=\frac{1}{L-1}\sum_{i=0}^{L-2}\frac{|c_{n}\left[i\right]-c_{n}\left[i+1\right]|}{2}.$$

This scalar counts how frequently the receiver-side switch state changes within one code period. It is not introduced as a complete description of the code spectrum; rather, it is introduced because it connects the discrete waveform design to the analog switching mechanism in a transparent way.

Let the first-difference sequence be $d_{n}\left[i\right]=\left(c_{n}\left[i+1\right]-c_{n}\left[i\right]\right)/2$. Since $d_{n}\left[i\right]\in \left\{-1,0,+1\right\}$,

(15) $$∥d_{n}{∥}_{2}^{2}=\sum_{i=0}^{L-2}{\left|d_{n}\left[i\right]\right|}^{2}=\left(L-1\right)\gamma_{n}.$$

Denote the discrete-time Fourier transforms of $c_{n}$ and $d_{n}$ by $C_{n}\left(e^{j\omega }\right)$ and $D_{n}\left(e^{j\omega }\right)$. Ignoring the single boundary term that vanishes for periodic extension, the difference relation gives

(16) $$D_{n}\left(e^{j\omega }\right)=\left(e^{j\omega }-1\right)C_{n}\left(e^{j\omega }\right).$$

Therefore, for any stopband region $\Omega_{h}=\left\{\omega :|\omega \right|\geq \omega_{0}\}$,

(17) $$\int_{\Omega_{h}}|C_{n}\left(e^{j\omega }\right){|}^{2}d\omega \leq \frac{1}{4\sin^{2}\left(\omega_{0}/2\right)}\int_{\Omega_{h}}{\left|D_{n}\left(e^{j\omega }\right)\right|}^{2}d\omega \leq \frac{2\pi \left(L-1\right)\gamma_{n}}{4\sin^{2}\left(\omega_{0}/2\right)}.$$

Equation (17) supplies the theoretical connection between the switching count and high-frequency code content: reducing $\gamma_{n}$ reduces an upper bound on the code energy available in high-frequency harmonic regions that can contribute to (8). The bound does not state that $\gamma_{n}$ uniquely determines $\alpha_{\mathrm{fold},n}$; two sequences with identical transition density may still have different line spectra. It justifies $\gamma_{n}$ as a dominant and physically interpretable design surrogate that must be verified by the exact PSD-based metric in (12).

A complementary statistical interpretation is obtained by approximating $c_{n}\left[i\right]$ as a first-order Markov sequence:

(18) $$R_{c_{n}}\left[m\right]=E\left\{c_{n}\left[i\right]c_{n}\left[i+m\right]\right\}={\left(1-2\gamma_{n}\right)}^{\left|m\right|}.$$

As $\gamma_{n}\to 0.5$, $R_{c_{n}}\left[m\right]$ approaches a delta-like correlation and the PSD envelope becomes flatter. As $\gamma_{n}$ decreases, the sequence becomes more slowly varying, concentrating more energy near low sequences and reducing the high-frequency content that drives broadband folding. This explains why conventional high-entropy PRN design, although useful for communication spreading and shifted correlation control, is not necessarily optimal for an internal receiver-side SSDBF address code.

The above discussion defines the hardware-noise side of the design problem. The next step is to examine the spatial-separation side. In the absence of timing mismatch, zero-lag orthogonality alone is sufficient, and the lowest-transition orthogonal reference can be identified analytically. This ideal case provides a useful benchmark, but it must later be modified for receiver-side timing skew.

### 3.2. Ideal Synchronization and the Walsh Lower Bound

Before introducing hardware timing mismatch, it is useful to isolate the ideal synchronized limit. If the analog code application and the digital demultiplexing operation are perfectly aligned, zero-lag orthogonality is sufficient for channel recovery. Under this assumption, the waveform design reduces to selecting an orthogonal subset with minimum aggregate transition density:

(19a) $$\begin{array}{cc} \min_{C_{\mathrm{sub}}\subset C_{\mathrm{univ}}}\; & \Gamma \left(C_{\mathrm{sub}}\right)=\sum_{n=1}^{K}\gamma_{n}, \end{array}$$

(19b) $$\begin{array}{cc} \mathrm{subject}\;\mathrm{to}\; & C_{\mathrm{sub}}C_{\mathrm{sub}}^{T}=LI_{K}. \end{array}$$

For a sequency-ordered Walsh–Hadamard matrix $H_{L}^{W}$, the transition density of the *r*-th row is $\gamma_{r}=r/\left(L-1\right)$ [22,23,24]. Thus, the ideal zero-delay lower bound is obtained by selecting the first *K* low-sequency rows:

(20) $$C_{\mathrm{sub}}^{\mathrm{opt}}=H_{L}^{W}\left[0:K-1,:\right].$$

This deterministic low-sequency Walsh selection is important because it separates two issues that are often coupled in conventional spreading-code design. On the one hand, it establishes the minimum switching-activity reference among orthogonal Walsh rows under perfect alignment. On the other hand, it also exposes the limitation of using zero-lag orthogonality as the only spatial-isolation criterion. Walsh orthogonality is a zero-shift property. Once there is a relative timing offset between analog code application and digital demultiplexing, non-zero-shift correlation becomes the relevant isolation metric.

The following subsection, therefore, moves from the ideal lower-bound reference to the practical quasi-synchronous receiver case. This transition is necessary because the proposed code set must not only reduce switching-induced noise but also preserve spatial separability when small receiver-internal timing offsets are present.

### 3.3. Quasi-Synchronous Misalignment and Shifted Correlation

The ideal Walsh lower bound in Section 3.2 assumes that the receiver applies and removes the analog address codes with exact chip-level alignment. In practical deployments of highly integrated micro-UAV radar sensors, this condition is difficult to maintain perfectly. Hardware nonidealities such as RF-switch control-path delay, PCB trace-length mismatch, FPGA or clock-tree skew, sampling-aperture uncertainty, and temperature-dependent delay variation can introduce fractional-chip timing offsets between analog modulation and digital demultiplexing. Under such quasi-synchronous conditions, zero-lag orthogonality alone no longer guarantees spatial channel isolation.

The timing error considered in this work is, therefore, an internal receiver-side misalignment. It is not primarily a transmitter-side synchronization problem. In the narrowband far-field model used here, wireless multipath may change the echo superposition, clutter structure, and received signal amplitude, but it does not directly create a relative code shift between two receive-channel address tags unless the signal bandwidth and channel dispersion become large enough to violate the narrowband assumption. This scope clarification is included to distinguish the present receiver-internal code-alignment problem from general propagation-channel equalization.

With a timing offset, the recovered channel contains shifted cross-products:

(21) $${\tilde{r}}_{p}=\int_{0}^{LT_{c}}\left[s_{p}\left(t\right)c_{p}\left(t\right)+\sum_{q\neq p}s_{q}\left(t\right)c_{q}\left(t-\tau_{\mathrm{skew}}\right)\right]c_{p}\left(t-\Delta \tau_{\mathrm{int}}\right)dt.$$

Here, $\Delta \tau_{\mathrm{int}}$ denotes the internal timing offset between the local demultiplexing code and the analog code applied to the desired channel, while $\tau_{\mathrm{skew}}$ represents the relative skew that exposes the interfering channel to a shifted code product. Equation (21) shows why the design cannot rely only on the zero-shift product $C_{\mathrm{sub}}C_{\mathrm{sub}}^{T}$. Walsh sequences are perfectly orthogonal at zero shift but can exhibit large off-zero-shifted correlation. Gold/PRN-like sequences have bounded shifted correlation, but their transition densities are typically close to 0.5, which increases switching and folded-noise penalties. Therefore, the design objective must balance the two properties rather than optimize either one alone.

The jitter-aware constraint is written as

(22) $$\max_{\tau \neq 0,\;p\neq q}\left|\sum_{i=0}^{L-1}c_{p}\left[i\right]c_{q}\left[i-\tau \right]\right|\leq \epsilon_{\mathrm{th}},\;\forall \;c_{p},c_{q}\in C_{\mathrm{sub}}.$$

This constraint should be interpreted as an engineering spatial-isolation requirement under receiver-side code misalignment, not as a claim of perfect orthogonality for all possible propagation delays. It preserves the role of shifted-correlation robustness while allowing the transition density to be reduced below the level typical of conventional PRN-like codes. Related bounded-correlation sequence constructions and combinatorial set-design principles provide useful context for this constrained selection problem [25,26].

Combining the hardware-noise objective from Section 3.1 with the shifted-correlation requirement yields the complete waveform-layer problem:

(23a) $$\begin{array}{cc} \min_{C_{\mathrm{sub}}\subset C_{\mathrm{univ}}}\; & \Gamma \left(C_{\mathrm{sub}}\right)=\sum_{n=1}^{K}\gamma_{n}, \end{array}$$

(23b) $$\begin{array}{cc} \mathrm{subject}\;\mathrm{to}\; & \max_{\tau \neq 0,\;p\neq q}\left|\sum_{i=0}^{L-1}c_{p}\left[i\right]c_{q}\left[i-\tau \right]\right|\leq \epsilon_{\mathrm{th}}. \end{array}$$

The formulation shows the central trade-off of the paper: low-transition-density sequences reduce the analog switching and folding burden, but the selected set must still survive receiver-side timing skew. This motivates the offline search strategy described next.

### 3.4. Search Strategy, Complexity, and Isolation Bound

The feasible subset is sparse because low-transition-density sequences tend to have poor shifted correlation, whereas shifted-correlation-friendly PRN sequences tend to switch frequently. The proposed ϵ-GCSS algorithm resolves this trade-off by generating a large candidate pool biased toward low transition density, sorting the candidates by γ, and greedily extracting the first subset that satisfies the shifted-correlation bound. The complete offline procedure is summarized in Algorithm 1.

The generation stage is $O(N_{\mathrm{total}}L)$. The greedy extraction stage is bounded by $O(KN_{\mathrm{total}}LN_{\tau })$, where $N_{\tau }$ is the number of evaluated shifts. Since sequence synthesis is performed offline and the selected code book is stored in the receiver, the algorithm does not impose real-time computational overhead on the micro-UAV radar.

The shifted-correlation threshold is selected as a pairwise spatial-isolation survival criterion rather than as a claim of perfect orthogonality under all possible delays. For a selected code set S, define

(24) $$\epsilon \left(S\right)=\max_{\tau \neq 0,\;p\neq q}\left|\sum_{i=0}^{L-1}c_{p}\left[i\right]c_{q}\left[i-\tau \right]\right|,\;\eta_{\mathrm{xtalk}}\left(S\right)=20\log_{10}\frac{\epsilon (S)}{L}.$$

For the main L=32 simulations, the threshold $\epsilon_{\mathrm{th}}\leq 11$ corresponds to $\eta_{\mathrm{xtalk}}\leq -9.28$ dB. This value is used as an engineering isolation-survival threshold in the timing-skew experiments: candidates below the threshold are retained for DBF evaluation, whereas candidates above it are treated as vulnerable to receiver-side code misalignment.
**Algorithm 1** Jitter-Constrained Greedy Coordinate Space Search (ϵ-GCSS)**Require:** 
Code length *L*, multiplexing depth *K*, pool size $N_{\mathrm{total}}$, threshold set E**Ensure:** 
Candidate Pareto sequence sets P  1:Initialize P←∅  2:Generate $N_{\mathrm{total}}$ bipolar candidates using a biased Markov transition probability $\gamma_{\mathrm{bias}}=\mu U^{2}$, U∼U(0,1)  3:Remove candidates violating balance or DC constraints if such constraints are required by the receiver  4:Sort the remaining candidates into $C_{\mathrm{sorted}}$ in ascending transition density  5:**for** each $\epsilon_{\mathrm{th}}\in E$ **do**  6:   Initialize $C_{\mathrm{sub}}\leftarrow \emptyset$  7:   **for** each candidate $c_{i}\in C_{\mathrm{sorted}}$ **do**  8:     Compute $\epsilon_{\max}=\max_{c_{p}\in C_{\mathrm{sub}},\;\tau \neq 0}\left|\sum_{t}c_{i}\left[t\right]c_{p}\left[t-\tau \right]\right|$  9:     **if** $\epsilon_{\max}\leq \epsilon_{\mathrm{th}}$ **then**10:        $C_{\mathrm{sub}}\leftarrow C_{\mathrm{sub}}\cup \left\{c_{i}\right\}$11:     **end if**12:     **if** $|C_{\mathrm{sub}}|=K$ **then**13:        $P\leftarrow P\cup \{C_{\mathrm{sub}}\}$ and stop the inner loop14:     **end if**15:   **end for**16:**end for**17:**return** P

## 4. Results

The preceding sections established the receiver-side noise-folding model and converted it into a waveform-layer design problem: the selected address-code set should reduce switching-related spectral content while preserving channel isolation under receiver-side timing skew. The numerical evaluation follows the same logic. It first verifies the circuit-level switching mechanism, then evaluates PSD-integrated in-band noise, compares representative code families, examines timing-skew and parameter sensitivity, and finally maps the resulting noise reduction to detection probability and normalized maximum range.

The transient-current and PSD results are generated from a commercial circuit-level transient simulation of an ADRF5020-class switch followed by MATLAB R2024b post-processing. This stage is used to isolate the code-dependent switching and spectral mechanisms under fixed device, control-edge, and loading conditions. It is followed by system-level MATLAB R2024b simulations using the same code-set metrics, namely mean transition density, worst shifted cross-correlation, calibrated folding factor, equivalent noise factor, probability of detection, and normalized maximum detection range. This validation chain is intended to make the waveform-dependent mechanism traceable from hardware switching behavior to radar-level performance, while leaving board-specific prototype qualification as a subsequent deployment step.

### 4.1. Simulation Setup and Validation Matrix

The simulated payload is a Ku-band micro-UAS radar operating at $f_{c}=15.0$ GHz. The nominal array has N=32 elements and uses K=4 as the main SSDBF multiplexing depth. To test generality beyond a single operating point, the simulations sweep *K*, *L*, $B_{\mathrm{IF}}$, chip rate, receiver-side timing skew, finite switch isolation, mutual coupling, calibration error, and code-family selection. Gold/PRN-like comparisons use length-compatible pseudo-random families when algebraic sequence lengths do not exactly match the Walsh length, and Kasami-type sequences are included as auxiliary algebraic benchmarks rather than as forced same-length replacements.

For the main L=32 timing-skew study, the pairwise isolation-survival threshold is $\epsilon_{\mathrm{th}}\leq 11$, corresponding to

(25) $$20\log_{10}\left(\frac{11}{32}\right)=-9.28\;\mathrm{dB}.$$

This threshold is not used to claim that Walsh, PRN, or ϵ-GCSS sequences are universally optimal. It provides a common receiver-side skew criterion under which zero-lag orthogonality, shifted-correlation robustness, and transition-density reduction can be compared. The complete system, hardware, and waveform configuration is summarized in Table 2.

**Table 3:** ADRF5020-class RF-switch parameters used in the circuit-level transient setup.

Item	Value or Modeling Use
Switch basis	ADRF5020-class silicon SPDT switch with nonreflective 50 Ω RFC/RF1/RF2 ports; used as a compact commercial Ku-band switch reference [27].
Frequency coverage	Datasheet range 100 MHz–30 GHz, covering the 15 GHz carrier used in the simulations.
Insertion loss and matching	1.5 dB typical RFC–RF1/RF2 insertion loss and 16 dB on-state return loss in the 10–20 GHz band. These values define the matched switch path used in the circuit-level setup.
Isolation	60 dB RFC–RF1/RF2 isolation and 65 dB RF1–RF2 isolation in the 10–20 GHz band. The nonideality study varies isolation around this nominal range.
Switching timing	2 ns RF-output rise/fall time, 10 ns on/off time, and 15 ns RF settling time to 0.1 dB; these values define the finite switching edge and the timing window used in the transient study.
Control and supply	$V_{\mathrm{DD}}=3.3$ V, $V_{\mathrm{SS}}=-2.5$ V, and $V_{\mathrm{CTRL}}=0/3.3$ V, consistent with CMOS/LVTTL-compatible control and dual-supply operation.
Power-handling check	18 dBm hot-switching recommended limit. The simulated receive-side switching level is kept below this rating, so large-signal compression is not the limiting mechanism in the reported noise-folding study.
Transient charge/feedthrough	The datasheet does not provide a standalone charge-injection coefficient. The switching-noise contribution is, therefore, evaluated from exported time-domain current/voltage waveforms, cumulative injected charge, and PSD integration rather than from an assumed scalar charge-injection constant.

### 4.2. Circuit-Level Validation of Hardware-Induced Noise Mapping

The first validation step examines whether the transition-density difference between code families produces distinguishable switching behavior at the RF multiplexing node. To keep this comparison tied to a realistic receive front-end, the circuit-level switching stage uses an ADRF5020-class silicon SPDT model [27]. At the 15 GHz carrier used here, the datasheet-supported operating point is consistent with a 10–20 GHz switch model: approximately 1.5 dB insertion loss, 60 dB RFC-to-throw isolation, 65 dB throw-to-throw isolation, 2 ns RF rise/fall time, and 15 ns RF settling time to 0.1 dB. These values make the circuit-level setup consistent with the Ku-band SSDBF case.

**Figure 2:**
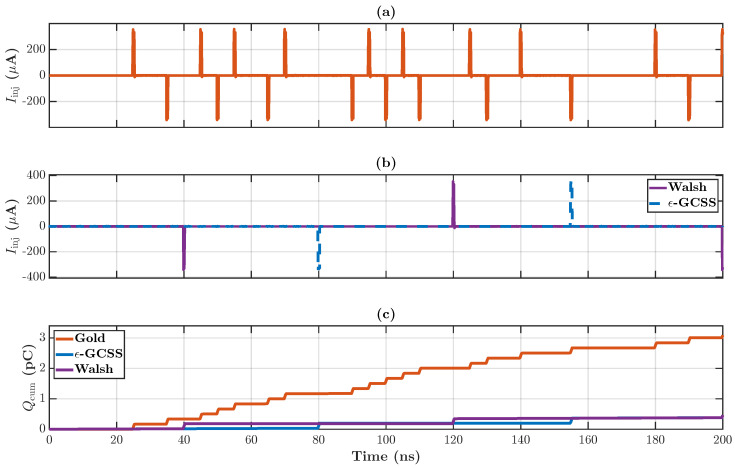
Circuit-level transient validation of switching-current injection: (**a**) transient current generated by the Gold/PRN-like code; (**b**) transient currents generated by the low-sequency Walsh and proposed ϵ-GCSS codes; and (**c**) cumulative injected charge for the three code families. The Gold/PRN-like code produces denser switching impulses, whereas Walsh and ϵ-GCSS produce fewer switching events and lower cumulative injected charge.

Within this controlled setup, all device and loading parameters are fixed and only the address-code family is varied. The exported transient current waveform and the integrated charge in Figure 2 are, therefore, used to evaluate the code-dependent switch-transient contribution. The term “charge injection” is used in this behavioral sense; it is not introduced as an independent datasheet scalar.

Figure 2 compares the circuit-simulated transient current and cumulative injected charge for Gold/PRN-like, Walsh, and ϵ-GCSS sequences. The Gold/PRN-like waveform produces dense switching impulses and a substantially larger cumulative charge, whereas Walsh and ϵ-GCSS produce fewer switching events. This result supports the separate switch-transient term in (10) and (11): transition density does not merely change an abstract code metric, but changes how often the RF switching network is excited.

**Figure 3:**
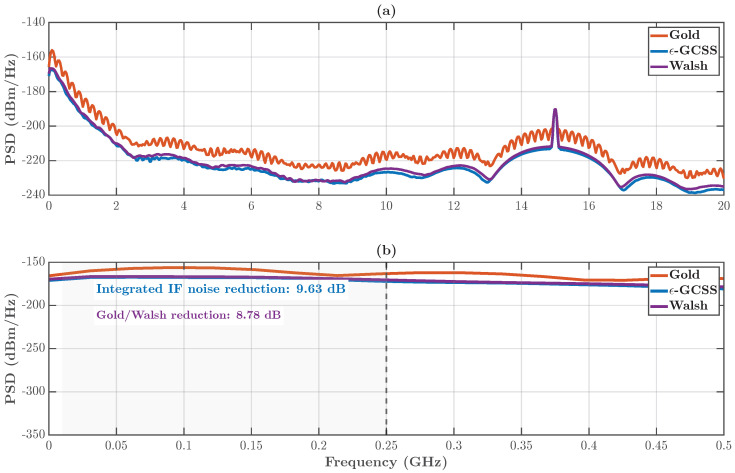
Circuit-simulation-derived PSD validation of switching-noise reduction: (**a**) wideband PSD comparison among the Gold/PRN-like, low-sequency Walsh, and proposed ϵ-GCSS codes; and (**b**) enlarged PSD view over the shared IF band. The dashed vertical line marks the upper integration boundary $B_{\mathrm{IF}}/2$. The in-band PSD integration gives a 9.63 dB reduction from the Gold/PRN-like baseline to the proposed ϵ-GCSS code set.

Figure 3 evaluates the corresponding circuit-simulation-derived power spectral densities after MATLAB Welch processing. The in-band comparison is obtained by integrating the PSD within the shared IF band:

(26) $$\Delta P_{\mathrm{noise}}=10\log_{10}\frac{\int_{0}^{B_{\mathrm{IF}}/2}S_{\mathrm{Gold}}\left(f\right)df}{\int_{0}^{B_{\mathrm{IF}}/2}S_{\epsilon \text{-}\mathrm{GCSS}}\left(f\right)df}.$$

The transient simulation data give $\Delta P_{\mathrm{noise}}=9.63$ dB for the Gold/PRN-like to ϵ-GCSS comparison, and 8.78 dB for the Gold/PRN-like to low-sequency Walsh comparison. The value should, therefore, be interpreted as a circuit-simulation-derived, PSD-integrated IF-noise reduction obtained under a fixed switch model and processing pipeline.

**Figure 4:**
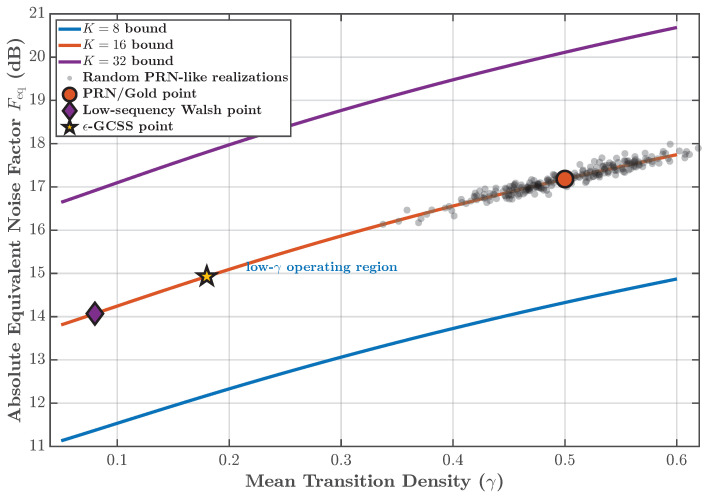
Absolute equivalent noise factor versus mean transition density. The three curves correspond to K=8, K=16, and K=32; the PRN/Gold, low-sequency Walsh, and ϵ-GCSS points are representative nominal operating points.

Figure 4 summarizes the transition-density dependence of the absolute equivalent noise factor. The figure explicitly plots the K=8, K=16, and K=32 bounds so that the role of multiplexing depth is unambiguous. For a fixed *K*, increasing γ increases $F_{\mathrm{eq}}$; for a fixed γ, increasing *K* raises the absolute equivalent-noise penalty. The PRN/Gold point lies in the high-transition-density region, whereas the ϵ-GCSS point moves toward the low-γ operating region while remaining on the nominal K=16 evaluation curve.

These circuit-level and system-level trends are consistent with the theoretical explanation in Section 3.1. Lower transition density reduces an upper bound on high-frequency code energy and lowers switching-event count, while the final noise penalty is still determined by PSD integration and the receiver IF response rather than by γ alone.

### 4.3. Code-Family Benchmark, Timing-Skew Robustness, and Parameter Sensitivity

**Figure 5:**
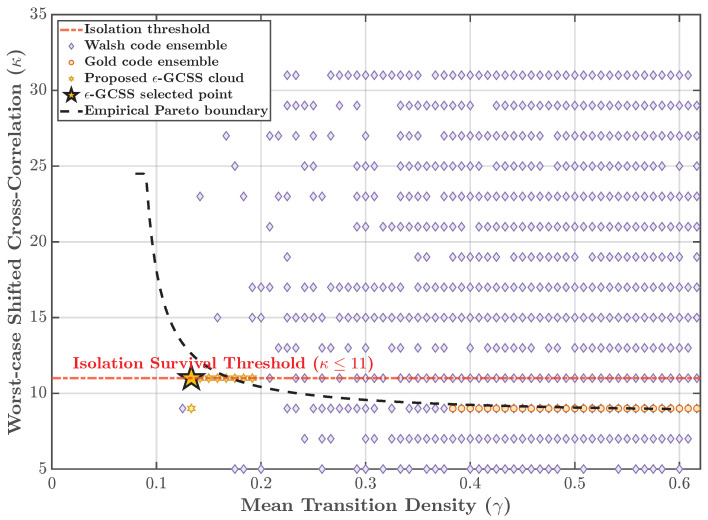
Transition-density and shifted-correlation Pareto frontier for SSDBF code-set selection.

Figure 5 shows the trade-off between mean transition density and worst-case shifted cross-correlation. Walsh sequences occupy a low-transition-density region but do not generally provide shift-invariant isolation. Gold/PRN-like sequences provide better non-zero-shift behavior but cluster near γ≈0.5. The proposed ϵ-GCSS candidate lies near the empirical Pareto boundary by reducing transition density while satisfying the imposed pairwise isolation threshold.

**Figure 6:**
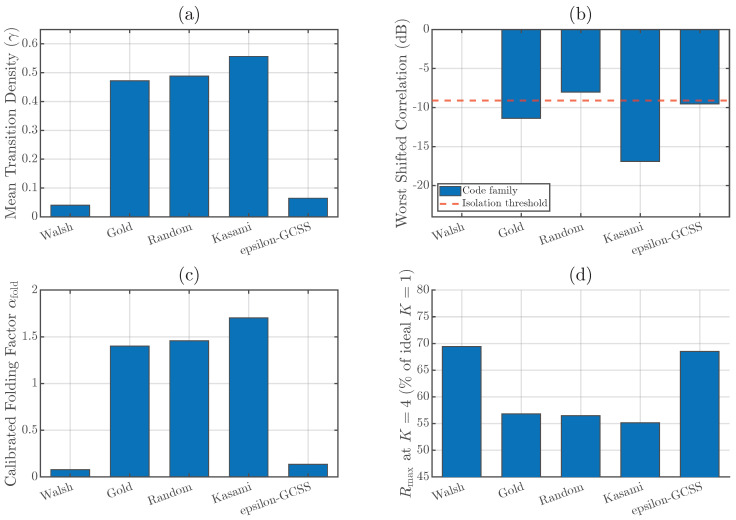
Expanded code-family benchmark: (**a**) mean transition density; (**b**) worst shifted cross-correlation; (**c**) calibrated folding factor; and (**d**) normalized maximum detection range for Walsh, Gold/PRN-like, random binary, Kasami/compatible PRN, and ϵ-GCSS sequences under the same SSDBF evaluation pipeline.

**Figure 7:**
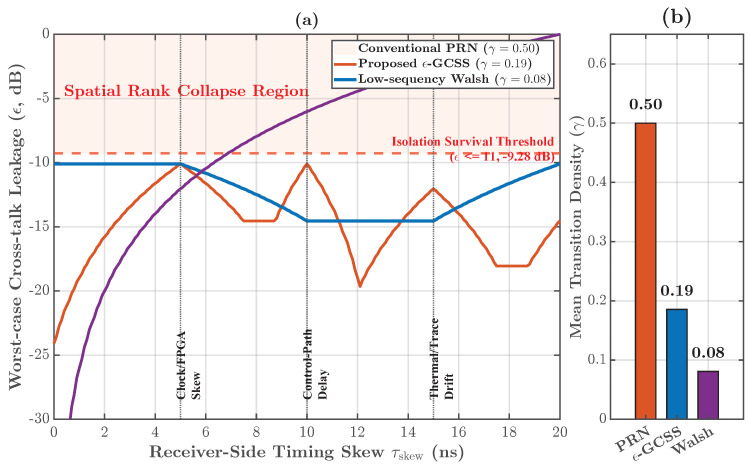
Code-set robustness under receiver-side timing skew: (**a**) worst-case cross-talk leakage versus receiver-side timing skew, where the dashed line denotes the isolation-survival threshold $\epsilon_{\mathrm{th}}=11$ (−9.28 dB) and the shaded region denotes spatial-rank collapse; and (**b**) mean transition-density comparison for the PRN, ϵ-GCSS, and low-sequency Walsh code sets. Walsh orthogonality is a zero-shift property, whereas the proposed ϵ-GCSS code set remains below the prescribed isolation threshold over the evaluated skew region.

Table 4 and Figure 6 provide the expanded code-family benchmark. The same evaluation pipeline is applied to Walsh, Gold/PRN-like, random binary, Kasami/compatible PRN, and ϵ-GCSS sequences. Walsh is included as a zero-shift lower-bound reference; its timing-skew vulnerability is evaluated separately in Figure 7. The benchmark, therefore, avoids treating any single code family as universally superior and instead reports the trade-off among transition density, shifted correlation, folding factor, and range.

Figure 7 evaluates the receiver-side timing-skew model introduced in Section 3.3. The purpose of this experiment is to test whether the code set that is favorable for switching-noise suppression also survives non-zero-shift channel leakage. Walsh remains a useful ideal-alignment reference, but its zero-lag orthogonality does not guarantee isolation under arbitrary non-zero shifts. The proposed ϵ-GCSS set is, therefore, judged by the same worst-case shifted-correlation threshold used in the synthesis stage. The present figure reports a deterministic skew sweep; a full statistical jitter model would require a hardware-specific clock-tree distribution and is left as a deployment-specific extension.

**Figure 8:**
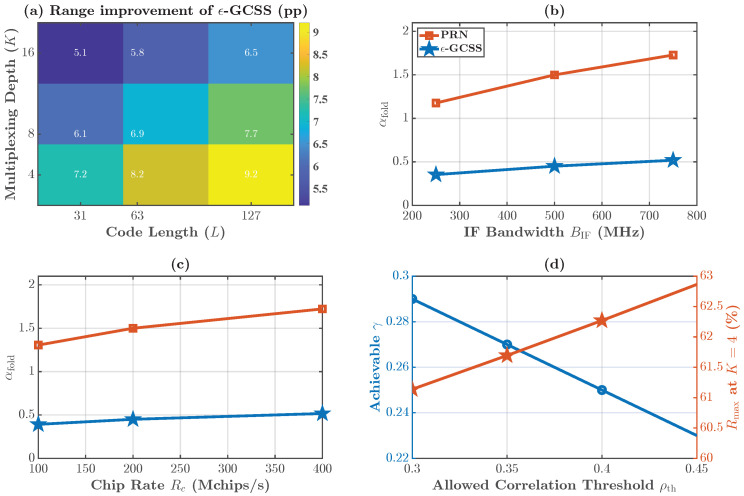
Parameter sensitivity: (**a**) range-improvement heatmap versus multiplexing depth *K* and code length *L*; (**b**) calibrated folding factor versus IF bandwidth $B_{\mathrm{IF}}$; (**c**) calibrated folding factor versus chip rate $R_{c}$; and (**d**) achievable transition density and normalized maximum range versus the isolation threshold $\epsilon_{\mathrm{th}}$.

Figure 8 reports the parameter-sensitivity study. The range-improvement heatmap shows that the ϵ-GCSS advantage persists across the evaluated *K* and *L* combinations, although the percentage-point gain becomes smaller at larger multiplexing depth. The $B_{\mathrm{IF}}$ and $R_{c}$ sweeps show that the folding factor grows when more folded spectrum is admitted by the receiver or when faster switching excites a wider spectral region. The $\epsilon_{\mathrm{th}}$ sweep shows the expected trade-off: relaxing the shifted-correlation constraint allows lower transition density and a higher normalized range, but at the cost of weaker isolation robustness.

### 4.4. End-to-End Detection, Range, and Nonideality Trade-Offs

The maximum detection range is first mapped by substituting (13) into the radar range equation:

(27) $$R_{\max}=\sqrt[{4}]{\frac{P_{t}G_{t}G_{r}\sigma \lambda^{2}}{{\left(4\pi \right)}^{3}kT_{0}BF_{\mathrm{eq}}{\mathrm{SNR}}_{\min}}}.$$

For normalized comparison against an ideal non-multiplexed reference, the range is written as

(28) $$\tilde{R}\left(\gamma \right)={\left[K(1+{\bar{\alpha }}_{\mathrm{fold}}\left(\gamma \right))\right]}^{-1/4}.$$

At K=4, the PRN-like baseline uses ${\bar{\alpha }}_{\mathrm{fold}}=1.50$, yielding

(29) $${\tilde{R}}_{\mathrm{PRN}}={\left[4\left(1+1.50\right)\right]}^{-1/4}=56.2\%.$$

The proposed ϵ-GCSS case uses ${\bar{\alpha }}_{\mathrm{fold}}=0.45$, yielding

(30) $${\tilde{R}}_{\epsilon \text{-}\mathrm{GCSS}}={\left[4\left(1+0.45\right)\right]}^{-1/4}=64.4\%.$$

**Figure 9:**
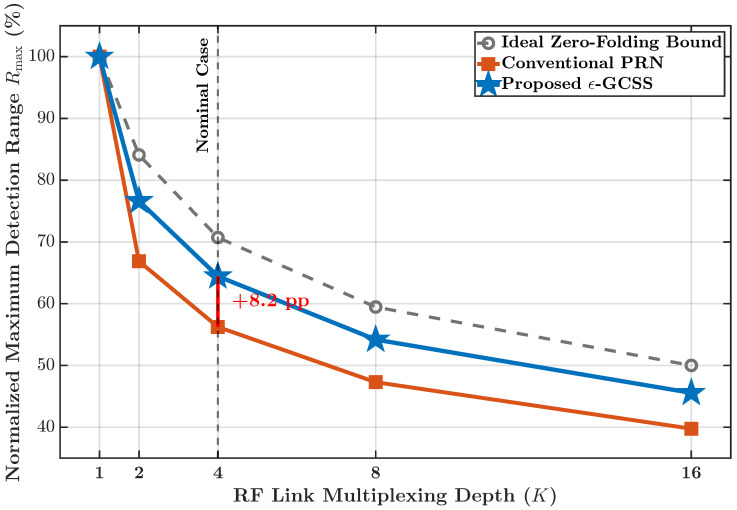
Normalized maximum detection range versus RF-link multiplexing depth. The proposed ϵ-GCSS code set recovers 8.2 percentage points at the nominal K=4 case relative to the PRN-like baseline.

Therefore, the range gain is 8.2 percentage points relative to the ideal non-multiplexed normalization, or approximately 14.6% relative to the PRN-like baseline. This range metric is a system-level radar-equation mapping and is reported separately from the 9.63 dB circuit-simulation-derived IF-noise reduction. Figure 9 summarizes the normalized range trends over the evaluated multiplexing depths.

**Figure 10:**
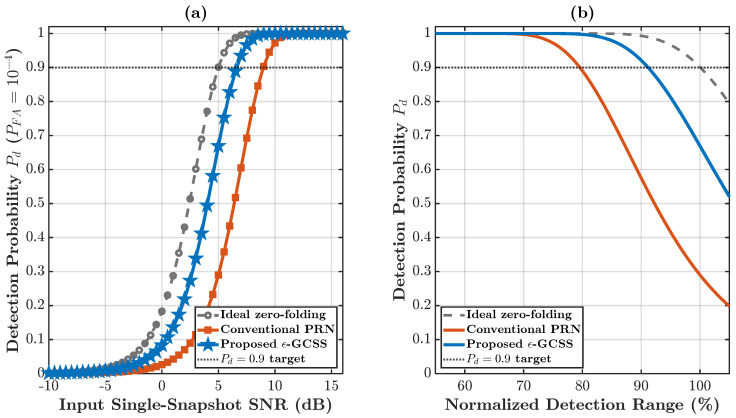
Detection-probability trade-off under a fixed false-alarm probability: (**a**) detection probability $P_{d}$ versus input single-snapshot SNR; and (**b**) $P_{d}$ versus normalized detection range. The proposed ϵ-GCSS code set improves both relationships relative to the conventional PRN baseline.

Figure 10 provides the $P_{d}/P_{\mathrm{FA}}$ interpretation. At the fixed $P_{\mathrm{FA}}=10^{-4}$ operating point, the proposed ϵ-GCSS curve lies between the ideal zero-folding reference and the conventional PRN baseline. Equivalently, for the same normalized range, ϵ-GCSS maintains a higher detection probability than PRN because less folded and switch-induced noise is admitted into the recovered spatial channels.

**Figure 11:**
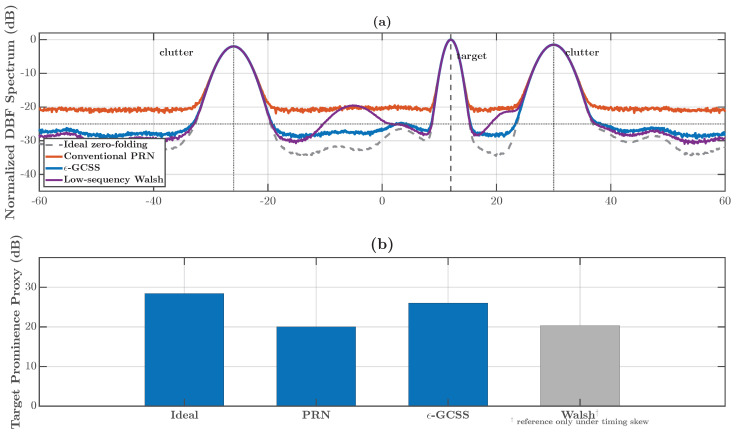
End-to-end radar-scene validation: (**a**) normalized DBF angular spectrum showing the target and clutter responses for the ideal zero-folding, conventional PRN, proposed ϵ-GCSS, and low-sequency Walsh cases; and (**b**) target-prominence proxy for the four cases. The Walsh result is included only as an ideal-alignment reference because its timing-skew behavior is assessed separately.

Figure 11 adds the end-to-end radar-scene validation. The simulation includes target echoes, clutter, receiver noise, quantization, demultiplexing, and DBF. The PRN-like case has a higher recovered noise floor, whereas ϵ-GCSS improves the target-prominence proxy and remains closer to the ideal zero-folding reference. The Walsh bar is retained as a zero-shift reference only; timing-skew robustness is evaluated in Figure 7.

**Figure 12:**
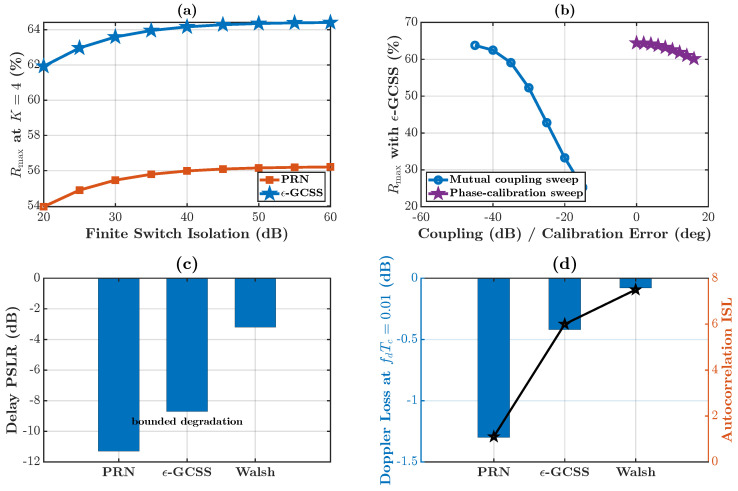
Practical nonideality and radar-metric sensitivity: (**a**) normalized maximum range versus finite switch isolation; (**b**) normalized maximum range under mutual-coupling and phase-calibration errors; (**c**) delay-domain peak sidelobe ratio and autocorrelation integrated sidelobe level for the compared code families; and (**d**) Doppler loss at the specified normalized Doppler offset.

Figure 12 summarizes additional nonideality and radar-metric checks. Finite switch isolation limits the achievable range, but ϵ-GCSS remains above the PRN baseline across the isolation sweep. Mutual coupling and calibration errors reduce performance as expected. The PSLR/ISL and Doppler-loss panels indicate that the proposed receive-side address-code design does not create an unbounded degradation in standard radar metrics over the tested conditions. These checks are important because transition density is a hardware-aware surrogate, not a complete radar-performance descriptor. The figure is intended as a representative sidelobe and Doppler-tolerance check; a full ambiguity-function optimization is outside the scope of the present receiver-address-code study and is left to future radar-waveform co-design.

The extended validation set shows how the proposed waveform design should be interpreted. Reducing γ is valuable because it lowers high-frequency code energy and switching-event count, and the circuit-simulation-derived PSD confirms the resulting in-band noise reduction. However, final code selection must also pass shifted-correlation, parameter-sensitivity, detection-probability, end-to-end radar-scene, and nonideality checks before deployment.

## 5. Discussion

The results above indicate that transition-density reduction is useful only when it is interpreted within the complete SSDBF receiver chain. It is not a standalone descriptor of folded noise for all possible codes and hardware implementations. The exact modulation-induced folding term depends on the harmonic coefficients $|A_{n,k}{|}^{2}$, and the switch-transient term depends on device response g(t), parasitic capacitance, control feedthrough, rise/fall time, package parasitics, and layout-dependent coupling. In this sense, the role of γ is to provide a tractable hardware-aware design surrogate: it upper-bounds high-frequency code energy through the first-difference relation and scales the number of switching events that excite device transients. The final candidate is, therefore, not accepted by transition density alone, but by PSD integration, shifted-correlation tests, detection-probability curves, radar-scene simulation, and nonideality checks.

The applicability of the proposed design also depends on the receiver topology. The LNA-less and preselector-limited architecture considered in this paper represents an extreme SWaP-C boundary case in which the multiplexing operation is exposed directly to antenna-port noise and switch-induced transients before shared amplification and filtering. If a particular platform can tolerate moderate per-element gain or compact preselection, analog filtering and waveform-level mitigation should be co-designed rather than treated as mutually exclusive solutions. Conversely, when high-Q preselection or per-element LNAs are not compatible with the payload budget, the internal address code becomes one of the few available design variables for reducing impairment before the shared receiver chain.

The timing-skew results should be read in the same receiver-side context. Section 3.3 treats the timing error as an internal mismatch between analog code application and digital demultiplexing, caused by clock distribution, switch-control delay, trace mismatch, sampling-aperture uncertainty, and thermal drift. This differs from transmitter synchronization and from wireless multipath. Under the narrowband far-field assumption used in this work, multipath changes the echo and clutter superposition, but it does not directly impose a different internal code shift on each receive-channel address tag. Wideband dispersive channels would require an extended model in which propagation-induced delay spread and receiver-internal code misalignment are treated jointly.

The circuit-level and system-level simulations are intended to provide a controlled hardware-waveform validation path before fabricated prototype testing. A hardware prototype would include one particular switch, driver, package, PCB stack-up, clock tree, grounding scheme, and ADC, so the measured impairment would contain both waveform-dependent and board-specific effects. By fixing the ADRF5020-class switch parameters and varying only the code family, the present circuit-level transient study isolates the waveform-dependent part of the folding and transient-injection mechanism. Prototype measurements remain necessary to determine the final proportionality between transition density, folded-noise power, and switch-transient injection in a specific implementation.

Scalability is another practical consideration. The ϵ-GCSS algorithm is offline and does not impose real-time processing load, but the feasible low-transition-density region can become sparse when *K* increases or when $\epsilon_{\mathrm{th}}$ is tightened. Larger arrays may require hierarchical grouping, structured low-transition-density generators, or hybrid algebraic-random constructions. These extensions are compatible with the present framework because the acceptance metrics remain unchanged: transition density, shifted-correlation survival, PSD-integrated noise reduction, and radar-level detection performance.

## 6. Conclusions

This paper presented a hardware-waveform co-design framework for SSDBF receivers on extreme SWaP-C radar platforms. The analysis separated modulation-induced thermal-noise folding from switch-transient injection and showed how both mechanisms are influenced by the spectral and switching properties of internal bipolar address codes. A first-difference spectral bound was derived to explain why reducing transition density suppresses high-frequency code energy, while also clarifying that transition density is a design surrogate rather than a sufficient statistic for all folded noise.

To exploit this relationship, an ϵ-GCSS algorithm was developed to synthesize low-transition-density code sets subject to a worst-case shifted-correlation constraint. The constraint accounts for receiver-side timing skew caused by clock distribution, switch-control delay, trace mismatch, and thermal drift. The resulting sequence set occupies the practical middle ground between low-sequency Walsh codes, which are efficient under ideal synchronization but shift-sensitive, and Gold/PRN-like codes, which have better shifted-correlation behavior but high transition density.

Using circuit-level transient data and MATLAB R2024b post-processing, the proposed ϵ-GCSS code set reduces the PRN-like transition density from about 0.50 to about 0.19 and lowers the integrated IF noise power by 9.63 dB relative to the Gold/PRN-like baseline. System-level radar simulations further show an 8.2-percentage-point improvement in normalized maximum detection range at K=4, improved $P_{d}$ behavior at fixed $P_{\mathrm{FA}}$, and better target prominence in an end-to-end radar scene. These gains are obtained without adding array-scaled analog front-end hardware. The result should be interpreted as a controlled hardware-waveform design study: the circuit-level model isolates code-dependent switching effects, whereas future device measurements and hardware-in-the-loop radar tests will quantify board-specific parasitics and deployment margins. Future work will combine the proposed waveform-level mitigation with device measurements, larger-array code construction, and hardware-in-the-loop radar validation.

## Figures and Tables

**Table 1:** Representative receiver power budget for a 32-element array.

Component	Conventional DBF	SSDBF (K=4)	Difference
High-speed ADCs	32×250 mW	8×250 mW	−6000 mW
SOI RF switches	0 mW	32×2 mW	+64 mW
FPGA I/O lanes	32×50 mW	8×50 mW	−1200 mW
Clock distribution	100 mW	150 mW	+50 mW
Total estimate	9.70 W	2.66 W	∼72.5% reduction

**Table 2:** System, hardware, and waveform parameters used in the simulation study.

Parameter	Value
Radar and platform parameters	
Carrier frequency ($f_{c}$)	15.0 GHz
Peak transmit power ($P_{t}$)	10 W
Signal bandwidth (*B*)	100 MHz
Base RF-chain noise figure ($F_{\mathrm{RF}}$)	3.5 dB
Target RCS (σ)	$1.0\;m^{2}$
SSDBF hardware and circuit-level transient parameters	
Array size (*N*)	32 elements
Main multiplexing depth (*K*)	4; sweeps: 1,2,4,8,16
Chip rate ($R_{c}$)	200 Mcps; sensitivity sweep included
IF bandwidth ($B_{\mathrm{IF}}$)	250–500 MHz
Timing skew ($\tau_{\mathrm{skew}}$)	0–20 ns deterministic receiver-side sweep
RF-switch model basis	ADRF5020-class silicon SPDT switch; key model parameters are listed in Table 3
Waveform, isolation, and radar-scene parameters	
Code length (*L*)	31, 32, 63, and 64 in sensitivity studies
Code families	Walsh, Gold/PRN-like, random binary, Kasami/compatible PRN, and ϵ-GCSS
Spatial isolation threshold ($\epsilon_{\mathrm{th}}$)	$\epsilon_{\mathrm{th}}\leq 11$ for the main L=32 case
End-to-end impairments	Clutter, thermal noise, ADC quantization, finite switch isolation, mutual coupling, and calibration error
Main metrics	γ, shifted correlation, PSD-integrated noise, $F_{\mathrm{eq}}$, $P_{d}/P_{\mathrm{FA}}$, and $\tilde{R}$

**Table 4:** Code-family benchmark under the common SSDBF metric pipeline.

Code Family	Metric Behavior	Role in This Work
Low-sequency Walsh	$\bar{\gamma }\approx 0.04$–0.08; ideal zero-shift orthogonality and low folding trend, but sensitive to non-zero shifted correlation under receiver-side skew.	Zero-shift lower-bound reference; not used as a stand-alone quasi-synchronous design.
Gold/PRN-like	$\bar{\gamma }\approx 0.47$–0.50; bounded non-zero-shift correlation, but high calibrated folding factor.	Conventional robust but noisy baseline; algebraic or PRN-like generation with negligible offline search burden.
Random binary	$\bar{\gamma }\approx 0.5$ on average; shifted correlation and folding factor vary across random draws.	Monte-Carlo reference for unstructured sequence selection.
Kasami/compatible PRN	Moderate-to-high transition density with bounded shifted-correlation behavior when a length-compatible construction is available.	Auxiliary algebraic comparison; not forced into incompatible lengths.
Proposed ϵ-GCSS	$\bar{\gamma }\approx 0.19$ in the main L=32 case; shifted correlation constrained by $\epsilon_{\mathrm{th}}$; low calibrated folding factor.	Main operating point; generated by offline greedy search with complexity $O(KN_{\mathrm{total}}LN_{\tau })$ and no real-time synthesis load.

The table summarizes the main L=32 operating point and the length-compatible family benchmark reported in the accompanying code-family comparison. Additional radar-metric checks are discussed later in the end-to-end validation subsection.

## Data Availability

The data presented in this study are available on request from the corresponding author. The data are not publicly available due to the ongoing research of this project.
